# Concepts for increasing gentamicin release from handmade bone cement beads

**DOI:** 10.3109/17453670903389782

**Published:** 2009-10-01

**Authors:** Hermawan N Rasyid, Henny C van der Mei, Henderik W Frijlink, Soegijardjo Soegijoko, Jim R van Horn, Henk J Busscher, Daniëlle Neut

**Affiliations:** ^1^Department of Biomedical Engineering, University Medical Center Groningen and the University of Groningenthe Netherlands; ^2^Biomedical Engineering Program, School of Electrical Engineering and Informatics, Institut Teknologi BandungIndonesia; ^3^Department of Pharmaceutical Technology and Biopharmacy, University of Groningen; ^4^Department of Orthopedic Surgery, University Medical Center Groningen and the University of Groningenthe Netherlands

## Abstract

**Background and purpose** Commercial gentamicin-loaded bone cement beads (Septopal) constitute an effective delivery system for local antibiotic therapy. These beads are not available in all parts of the world, and are too expensive for frequent use in others. Thus, orthopedic surgeons worldwide make antibiotic-loaded beads themselves. However, these beads are usually not as effective as the commercial beads because of inadequate release kinetics. Our purpose was to develop a simple, cheap, and effective formulation to prepare gentamicin-loaded beads with release properties and antibacterial efficacy similar to the commercially ones.

**Methods** Acrylic beads were prepared with variable monomer content: 100% (500 μL/g polymer), 75%, and 50% to increase gentamicin release through creation of a less dense polymer matrix. Using the optimal monomer content, different gel-forming polymeric fillers were added to enhance the permeation of fluids into the beads. Polyvinylpyrrolidone (PVP) 17 was selected as a suitable filler; its concentration was varied and the antibiotic release and antibacterial efficacy of these beads were compared with the corresponding properties of the commercial ones.

**Results** Gentamicin release rate and the extent of release from beads prepared with 50% monomer increased when the PVP17 content was increased. Beads with 15 w/w% PVP17 released 87% of their antibiotic content. This is substantially more than the gentamicin release from Septopal beads (59%). Acrylic beads with 15 w/w% PVP17 reduced bacterial growth by up to 93%, which is similar to the antibacterial properties of the commercial ones.

**Interpretation** A simple, cheap, and effective formulation and preparation process has been described for hand-made gentamicin-releasing acrylic beads, with better release kinetics and with antibacterial efficacy similar to that of the commercial ones.

## Introduction

In Europe, commercially available gentamicin-loaded polymethylmethacrylate beads (Septopal) constitute an effective delivery system for local antibiotic therapy for osteomyelitis (Bucholz and Engelbrecht 1970, Blaha et al. 1990, Klemm 1993), in combination with systemically delivered antibiotics and surgical debridement. The gentamicin concentrations reached at the site of infection are far higher using antibiotic-loaded bone cement beads than the concentrations achieved by systemic administration of the same antibiotic (Buchholz and Engelbrecht 1970), and far above the minimal inhibitory concentrations of most common pathogens (Wahlig et al. 1978). The use of antibiotic-loaded bone cement beads also gives very low antibiotic concentrations in serum and urine, thereby preventing toxic side effects (Diefenbeck et al. 2006). Alternatives to PMMA beads have been investigated, such as plaster of Paris beads (Dacquet et al. 1992, Bowyer and Cumberland 1994, Gaasbeek et al. 2005). Plaster of Paris is cheaper than bone cement and readily available, and small quantities can be used to make beads containing antibiotic. Plaster of Paris is tolerated when implanted into infected bone cavities, and is it absorbed over a period of weeks to months (Dacquet et al. 1992). Release rates from plaster of Paris beads are higher than from PMMA beads in the first 48 h (Bowyer and Cumberland 1994), but the release rates are much lower than from PMMA beads after this period. Since the PMMA beads work over a longer period of time (about 2 weeks), this paper will concentrate only on PMMA.

Gentamicin-loaded PMMA beads are not, however, commercially available in several parts of the world, including the USA, and they are too expensive for common use in many other countries of the world. Thus, orthopedic surgeons worldwide make antibiotic-loaded beads themselves, sometimes using a template system, but most often by hand-rolling. The antibiotic release kinetics from PMMA bone cements depend on the penetration of dissolution fluids into the polymer matrix and subsequent diffusion of the dissolved drug from the beads. Both steps require a certain porosity of the cement. Commercially prepared gentamicin-loaded acrylic beads are porous and show much higher release rates than hand-rolled, non-porous antibiotic-loaded acrylic beads (McLaren et al. 2004). Unfortunately, the exact method of pore production in these beads has not been disclosed.

In order to increase antibiotic release from hand-rolled acrylic beads, McLaren et al. (2004, 2006, 2007a) proposed the addition of soluble fillers, such as glycin, xylitol, sucrose, or erythritol in order to increase their porosity and consequently the penetration of the dissolution fluids. These soluble fillers have all increased the release of gentamicin from acrylic beads. Moreover, the amount of gentamicin release from an acrylic-glycine mixture increased with increasing amounts of glycine (McLaren et al. 2004). Furthermore, xylitol appeared to be more effective in increasing the amount of antibiotic release than glycine; for example, after 1 day, xylitol increased the release of daptomycin by a factor of 2.7 whereas glycine increased it 1.8 times as compared to beads without fillers (Mc Laren et al. 2006).

Although addition of these soluble fillers gave a doubling in antibiotic release from hand-rolled beads, the total release after 7 days remained limited to approximately 10% in the presence of soluble fillers, which is substantially inferior to the antibiotic release from commercially available beads (where the total release after 7 days amounts to around 60% of the total antibiotic content) (Walenkamp et al. 1986). Thus, the aim of this study was to develop a simple, cheap, and effective formulation and preparation process for acrylic beads with gentamicin release properties similar to those observed for commercially available beads. To this end, acrylic beads were first prepared with variable monomer content to give increased gentamicin release through the creation of a less dense polymer matrix. Subsequently, after the optimal monomer content had been defined, different gel-forming polymeric fillers such as polyvinylpyrrolidone (PVP) (of 2 different molecular weights) and hydroxypropylmethylcellulose (HPMC) were added to enhance the permeability for dissolution of fluids and gentamicin release. After selection of the most favorable biodegradable filler, its concentration was varied and both the antibiotic release and the antibacterial efficacy of the final beads were compared with the release and efficacy of the commercially available Septopal beads.

## Materials and methods

### Commercially available antibiotic beads

Gentamicin-loaded PMMA beads, which are commercially available under the name Septopal, were obtained from Biomet Europe (Darmstadt, Germany). One bead (7.0 mm in diameter) contains 7.5 mg of gentamicin sulfate.

### Preparation of beads with different concentrations of monomer

Simplex-P bone cement powder (Stryker Howmedica Osteonics; Howmedica International, Limerick, Ireland) was mixed with powdered gentamicin sulfate (Gracia Pharmaceutical, Indonesia) for 2 min in a ceramic bowl using a spatula. One gram of gentamicin sulfate was added to 40 g polymer powder. The resulting mixture was subsequently combined with 20 mL of monomer in a ceramic bowl and mixed for 2 min with a spatula according to the manufacturer's instructions. Beads thus prepared (500 μL/g polymer) will be termed “100% monomer”. In addition, beads were prepared with 75% and 50% of the prescribed amount of monomer (375 μL/g and 250 μL/g polymer, respectively). The material was mixed until a doughy paste was obtained, and the gentamicin-PMMA-MMA mixture was hand-rolled into beads.

### Preparation of beads with different polymeric fillers

Powdered PMMA and gentamicin sulfate (1 g gentamicin sulfate and 40 g PMMA powder) were mixed. Then one of the polymeric fillers was added to this powder mixture. 3 biocompatible gel-forming polymeric fillers were used: polyvinylpyrrolidone with a molecular weight of 360 kDa (PVP 90K) (Genfarma, Zaandam, the Netherlands), polyvinylpyrrolidone with a molecular weight of 7–11 kDa (PVP 17, Kollidon; BASF, Germany), and hydroxypropylmethylcellulose (HPMC; Sigma-Aldrich Chemie GmbH, Steinham, Germany). Polymeric fillers were mixed at a concentration of 10 w/w% with respect to the amount of polymer powder. The resulting mixtures were finally combined with 50% (250 μL/g polymer) of the prescribed amount of monomer and the beads were prepared as described above.

### Preparation of beads with different concentrations of PVP 17

Powdered PMMA and gentamicin sulfate (1 g gentamicin sulfate and 40 g PMMA powder) were subsequently mixed with different amounts of PVP 17 (5 w/w%, 10 w/w%, and 15 w/w% with respect to the amount of polymer powder) and beads were prepared with 50% monomer (250 μL/g polymer) as described above.

### Analysis of the release kinetics of gentamicin from the beads

A gentamicin-loaded acrylic bead was immersed in 10 mL sterile phosphate-buffered saline (PBS) at pH 7.4 and incubated at 37°C. At designated time intervals (6 and 24 h; and 2, 3, 7, and 14 days), 500-μL aliquots of the gentamicin solution in PBS were taken and the total amount of buffer was restored to 10 mL.

Gentamicin concentrations were measured using a procedure described by Sampath and Robinson (1990). Briefly, an o-phthaldialdehyde reagent was made and stored for 24 h in a dark environment. The gentamicin aliquot, o-phthaldialdehyde reagent, and isopropanol were mixed in equal proportions and stored for 30 min at room temperature. The o-phthaldialdehyde reacted with the gentamicin amino groups and chromophoric products were obtained, the absorbances of which were measured at 332 nm using a Genesys Spectronic 20 spectrophotometer (Spectronic Instruments, Rochester, NY). A calibration curve was used to calculate the gentamicin concentrations in the samples. The percentages of gentamicin released were calculated with respect to the total amount incorporated and compared with the gentamicin release from Septopal beads.

The release experiments with beads prepared with different concentrations of PVP 17 were performed in triplicate and a statistical analysis (Student's t-test for independent samples) was done in order to compare the gentamicin release rates from the handmade beads with those from commercial Septopal beads. A 95% (p < 0.05, 2-tailed) confidence interval was applied for statistical significance.

### Scanning electron microscopy

To compare the polymer matrix of our hand-rolled beads with that of Septopal beads, scanning electron microscopy (SEM) was performed. Examination was done at 2.0 kV in a JEOL field emission scanning electron microscope type 6301F. Beads were sputter-coated with a 3-nm thick conductive layer of gold/ palladium (80/20).

### Analysis of the antibacterial efficacy of the beads

3 hand-rolled or Septopal beads were each immersed in 5 mL tryptone soya broth (TSB; Oxoid Ltd., Cambridge, UK) and incubated at 37°C. Each bead was transferred on a daily basis to 5 mL fresh TSB and again incubated at 37°C, yielding broth containing antibiotic released over a 24-h period for biofilm growth studies. Only broth collected after 1, 2, 3, 7, and 14 days was used for further evaluation. Elution media were stored in a refrigerator at 4°C until further use.

Biofilm formation is a key process in the development and persistence of osteomyelitis, and *Staphylococcus aureus* is a common cause of osteomyelitis. Thus, biofilms were grown using a clinical strain, *S. aureus* 5298, isolated from a patient with an implant-related osteomyelitis at the University Medical Center Groningen, the Netherlands. This strain was gentamicin-sensitive, with an MIC value of 0.75 μg/mL. A preculture of the strain was used to fill 96-well plates with 200 μL bacterial suspension (2 μL preculture + 198 μL fresh TSB, or TSB collected as described above after antibiotic release from an immersed bead). Biofilms were grown for 24 h at 37°C. Subsequently, the wells were flushed with 200 μL PBS to remove free-floating bacteria. Then, the wells were stained with 200 μL 1% crystal violet for 30 min, washed with 200 μL demineralized water to remove the excess stain, and the crystal violet was solubilized in 200 μL of ethanol-acetone (80:20, v/v). The absorbance at 575 nm was subsequently determined using a microtiter plate reader and expressed as a percentage with respect to the absorbance of control biofilms grown in the absence of released antibiotics. All experiments were repeated 3 times with separately grown bacteria.

Statistical analysis was done in order to compare the antibacterial efficacy of the hand-rolled beads with that of Septopal beads. Differences were analyzed pair-wise for significance using Student's t-test, with significance assumed at p-values ≤ 0.05.

## Results

### Characteristics of the beads

The different hand-rolled beads had an average diameter of 13 mm and their average weight was 1.45 g, 1.34 g, and 1.23 g for beads prepared with 100%, 75%, and 50% monomer, respectively.

### Gentamicin release

**Figure 1.**
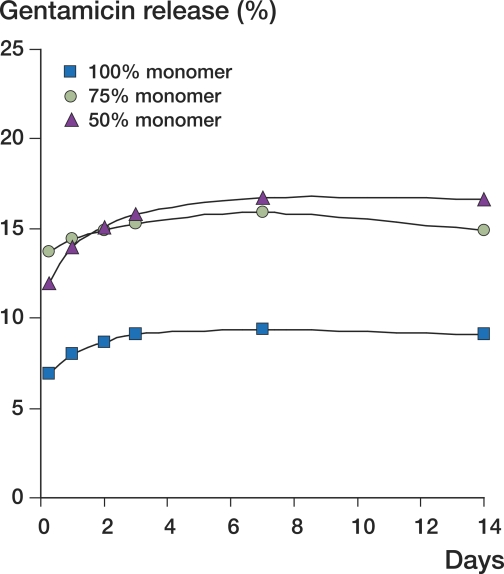
Cumulative percentage of gentamicin release from PMMA beads made with 100%, 75%, or 50% monomer, as a function of time during exposure to phosphate-buffered saline (PBS).

Release of gentamicin from beads prepared with 100% monomer leveled off within the time interval of the experiment, and was confined to about 8% of the total amount of gentamicin included. Reduction of the amount of monomer caused incomplete polymerization and sintering of the polymer beads, which resulted in an almost 2-fold increase in gentamicin release when 50% of monomer was employed, as compared to beads made with 100% monomer (Figure 1).

**Figure 2.**
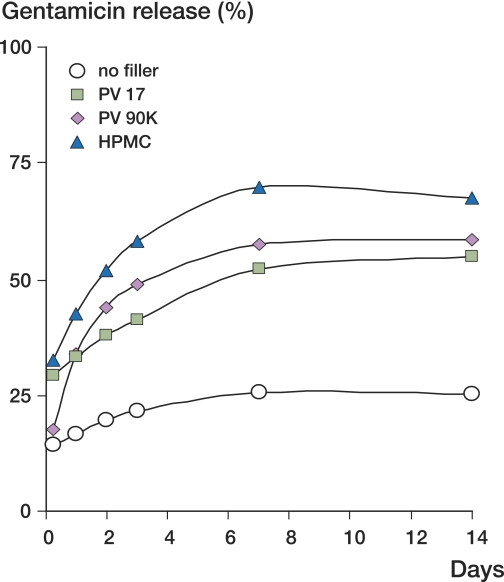
Cumulative percentage of gentamicin release from PMMA beads made without filler and with 10 w/w% of PVP 17, PVP 90K, or HPMC and 50% of the prescribed amount of monomer, as a function of time during exposure to PBS.

Inclusion of a biodegradable filler almost tripled the amount of gentamicin release compared to its release in the absence of fillers. There was little difference in release rates between the different fillers (Figure 2).

**Figure 3.**
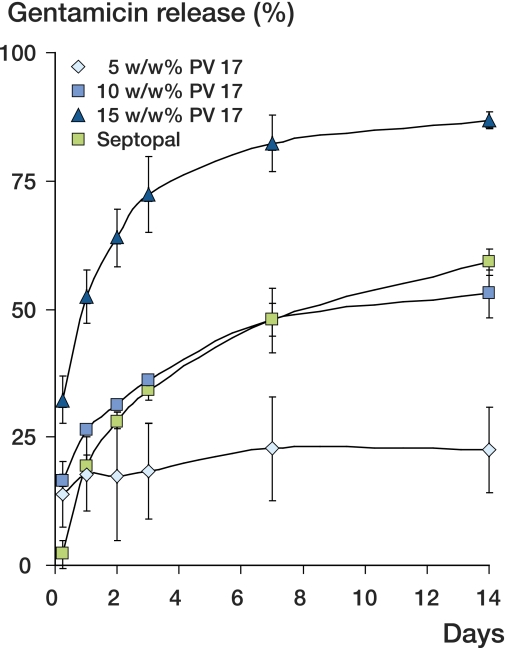
Cumulative percentage of gentamicin release from PMMA beads made with 5 w/w%, 10 w/w%, or 15 w/w% PVP 17 and 50% of the prescribed amount of monomer, as a function of time during exposure to PBS, in comparison to the gentamicin release from commercial Septopal beads. Error bars denote the SD for 3 different beads of hand-rolled and Septopal type.

Based on the observation that the dissolution of HPMC created a sticky and viscous medium, which was difficult to handle, and the fact that PVP 17 had the highest purity, we decided to vary the amount of PVP 17 (Figure 3) and to compare the gentamicin release kinetics of these cement beads with those of Septopal beads. The gentamicin release from beads prepared with 50% monomer increased when the amount of PVP 17 in the beads was increased. Beads containing 15 w/w% PVP 17 released 87% of their antibiotic content in 14 days. Importantly, this is significantly more than the gentamicin release from Septopal beads (p < 0.05), which is limited to only 59% after 14 days.

### Evaluation of bead porosities by SEM

**Figure 4.**
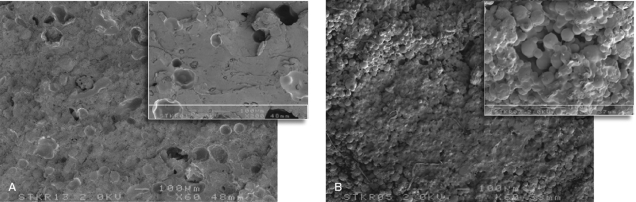
SEM micrographs of gentamicin-loaded, fractured acrylic beads: (A) prepared with the prescribed amount of monomer; (B) prepared with 50% of the prescribed amount of monomer. The images were taken from the fracture side of the beads. Scale bars represent 100 μm for low- and high-magnification micrographs and for the insert.

Beads prepared with 100% monomer formed a dense material with much mass and little porosity. Reduction of the amount of monomer relative to the prescribed amount caused a major increase in porosity, and the polymer particles appeared more “sintered” rather than “fused” together (Figure 4).

### Antibacterial efficacy

**Figure 5.**
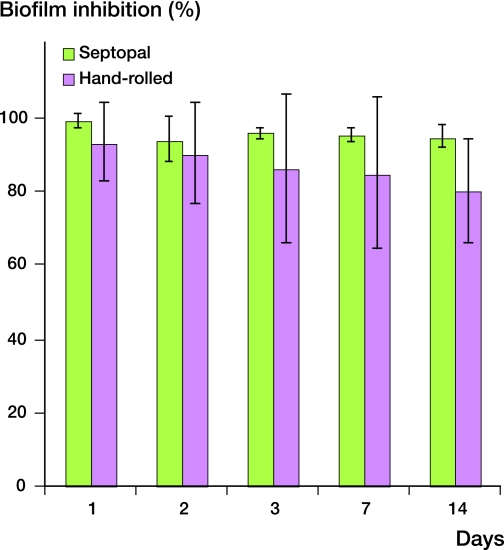
Biofilm inhibition (%) as a function of time, by different gentamicin-releasing beads, calculated as a percentage UV absorbance with respect to a control, i.e. the absorbance of a biofilm grown in broth without antibiotics released from beads. Bars represent the mean ± SD of triplicate results with separately grown bacteria.

Both bead systems showed high reductions in biofilm growth for up to 14 days after immersion (Figure 5). There was no statistically significant difference in biofilm inhibition between our hand-rolled beads and Septopal beads.

## Discussion

We have described a simple, cheap, and effective formulation and preparation process for gentamicin-releasing PMMA beads, with release kinetics that are somewhat better than those observed for commercially available Septopal beads. The improved release kinetics are mainly the result of increased porosity of the beads, due to the use of only 50% of the prescribed amount of monomer, which causes sintering rather than polymerization fusion of polymer particles, leaving a porous matrix. Secondly, the addition of a gel-forming, water-soluble polymeric filler, PVP 17, ensures penetration of fluids into all parts of the matrix (full percolation), thereby increasing the total amount of drug that is released.

The efficiency of the polymerization reaction became less when the proportion of liquid monomer added to the polymeric bone cement powder was reduced. If the amount of monomer is insufficient for the reaction to occur at all possible contact sites of the polymer particles, a more porous matrix will be formed, allowing penetration of water to most sites in the beads. This will lead to a higher amount of gentamicin being released (Frutos Cabanillas et al. 2000). On the other hand, with the reduction in the amount of monomer, the hardening time of the acrylic becomes considerably reduced (Pascual et al. 1999) and it requires some dexterity to prepare beads within the time available. Yet, it is possible to produce beads, although the last beads prepared out of a batch tend to appear brittle. In general, however, manual examination of the beads prepared with 50% monomer showed that they had sufficient strength for this non-load-bearing application of bone cement. Reduction of the monomer content to below 50% was impossible, as no coherence could be obtained at these lower concentrations (Willert et al. 1979).

The use of gel-forming, water-soluble polymeric fillers turned out to be indispensable to increase the gentamicin release to levels comparable to or higher than the release of gentamicin by the commercially available Septopal beads. Although we chose to use PVP 17 for this purpose, other biodegradable fillers might also have served the purpose. PVP 17-filled beads showed excellent gentamicin release profiles, and the Kollidon PVP 17 PF grade is considered safe for parenteral use in humans (Böhler 1999). Moreover, the PVP 17 could easily be combined with the acrylic polymer in the production process.

Several studies have been done to investigate the effect of biodegradable fillers on the drug release of antibiotic-loaded bone cement. Frutos et al. (2002) studied the influence of PVP 12 on the antibiotic release from commercially available gentamicin-loaded bone cement. Incorporation of PVP into the cement matrix led to a remarkable increase in the maximum amount of gentamicin released, and this effect was proportional to the PVP concentration incorporated. Virto et al. (2003) studied the antibiotic release of gentamicin-loaded bone cement after addition of HPMC. HPMC did not produce an increase in the gentamicin release and these authors hypothesized that dissolution of HPMC creates a surrounding similar to a gel that makes gentamicin release from the cement matrix difficult. However, we found a distinct increase in the gentamicin release after addition of HPMC to bone cement, with the clear distinction that our bone cement was made with 50% of the prescribed monomer. Also, McLaren et al. (2007a) investigated the use of biodegradable fillers but, like Frutos et al. (2002) and Virto et al. (2003), they did not use less monomer, and thus no additional intrinsic porosity was created (i.e. porosity achieved without dissolution of any filler material). In line with our findings, McLaren et al. (2007a,b) showed that it is not the filler material that is crucial but rather its particle size, and fillers with a larger particle size led to larger pores, less pore interconnectivity, and faster fluid penetration. Smaller-size particles led to smaller pores, greater pore interconnectivity, and smaller areas between the pores with no fluid penetration.

The gentamicin-loaded beads based on the use of half the prescribed amount of monomer and the addition of gel-forming polymeric filler reduced biofilm formation as effectively as commercially available Septopal beads. The (statistically insignificant) reduction in efficacy of our hand-rolled beads is presumably due to a less favorable area-to-volume ratio, as the hand-rolled beads were larger than the commercially available beads. Moreover, hand-rolled beads are usually not uniform in size, giving much larger standard deviations than obtained with commercially available beads and attesting to the greater reproducibility of Septopal beads. To solve this problem, a beads template system could be used, in which antibiotic-loaded beads of uniform size and small diameter could be produced. A template system should also be considered because of the short time available to prepare beads with half of the prescribed amount of monomer.

To verify the significance of our in vitro observations, in vivo studies will be required that take into account both complete eradication of the infection and the time required to achieve this outcome, but we believe the results reported here support our hypothesis that acrylic beads containing half the prescribed amount of monomer and PVP may offer a therapeutic efficacy similar to that of commercially available Septopal beads. Application of this novel formulation of acrylic beads in combination with a bead-template system would be useful for three different groups of orthopedic surgeons, to make chains of antibiotic-loaded bone cement beads: (1) surgeons in countries where they are not allowed to import the Septopal beads, such as the USA; (2) surgeons in developing countries, since these beads are approximately 3 times cheaper than the commercial ones (apart from the costs of the template system); and (3) surgeons who are familiar with the use of the Septopal beads but who need to treat their patients with another antibiotic because of the increasing number of patients infected with gentamicin-resistent bacteria. The method we describe here also makes it possible to create bone cement beads loaded with vancomycin, clindamycin, or other antibiotics that are not yet available commercially.

HR: concept, study design, data collection, analysis, and manuscript preparation. HvdM, HF, SS, JvH, and HB: study design, analysis, and revision of manuscript. DN: concept, study design, analysis, and revision of manuscript.

No competing interests declared.
